# Role of MicroRNA-182 in Posterior Uveal Melanoma: Regulation of Tumor Development through MITF, BCL2 and Cyclin D2

**DOI:** 10.1371/journal.pone.0040967

**Published:** 2012-07-27

**Authors:** Dongsheng Yan, Xiang Da Dong, Xiaoyan Chen, Shasha Yao, Lihua Wang, Jiao Wang, Chao Wang, Dan-Ning Hu, Jia Qu, LiLi Tu

**Affiliations:** 1 School of Ophthalmology and Optometry, Eye Hospital, Wenzhou Medical College, Wenzhou, Zhejiang, China; 2 State Key Laboratory Cultivation Base and Key Laboratory of Vision Science, Ministry of Health of the People’s Republic of China, Zhejiang Provincial Key Laboratory of Ophthalmology and Optometry, Wenzhou, Zhejiang, China; 3 Department of Surgery, Stamford Hospital – Affiliate of Columbia University, Stamford, Connecticut, United States of America; 4 Tissue Culture Center, New York Eye and Ear Infirmary, New York Medical College, New York, New York, United States of America; Duke University, United States of America

## Abstract

MicroRNAs (miRNAs) are endogenous small non-coding RNAs that play central roles in diverse pathological processes. In this study, we investigated the effect of microRNA-182 (miR-182) on the development of posterior uveal melanomas. Initially, we demonstrated that miR-182 expression was dependent on p53 induction in uveal melanoma cells. Interestingly, transient transfection of miR-182 into cultured uveal melanoma cells led to a significant decrease in cell growth, migration, and invasiveness. Cells transfected with miR-182 demonstrated cell cycle G1 arrest and increased apoptotic activity. Using bioinformatics, we identified three potential targets of miR-182, namely MITF, BCL2 and cyclin D2. miR-182 was shown to have activity on mRNA expression by targeting the 3′ untranslated region of MITF, BCL2 and cyclin D2. Subsequent Western blot analysis confirmed the downregulation of MITF, BCL2 and cyclin D2 protein expression. The expression of oncogene c-Met and its downstream Akt and ERK1/2 pathways was also downregulated by miR-182. Concordant with the findings that miR-182 was decreased in uveal melanoma tissue samples, overexpression of miR-182 also suppressed the *in vivo* growth of uveal melanoma cells. Our results demonstrated that miR-182, a p53 dependent miRNA, suppressed the expression of MITF, BCL2, cyclin D2 and functioned as a potent tumor suppressor in uveal melanoma cells.

## Introduction

Uveal melanoma is a tumor arising out of pigmented cells of the eye including the iris, ciliary body, or choroid [1]. Due to the behavioral and anatomical differences between the various types of uveal melanomas, all uveal melanomas with the exception of iris melanomas are collectively referred to as posterior uveal melanomas [2]. True iris melanoma, originating from within the iris as opposed to invasion from surrounding structures, is frequently associated with sun exposure similar to the much more common types of cutaneous melanoma [1]. Consequently, iris melanomas frequently harbor BRAF gene mutations associated with ultraviolet damage, and are less likely to metastasize than other uveal melanomas [3]. Posterior uveal melanomas behave similar to other non-sun exposure related melanomas, such as mucosal melanomas. Posterior uveal melanomas frequently harbor GNAQ mutations, but rarely BRAF mutations [4], [5]. These tumors behave aggressively and frequently present with hematogenous metastases to the liver early in the course of disease progression [1], [6].

The development of melanoma from a single melanocyte has been linked to a master regulator gene, the microphthalmia-associated transcription factor (MITF) [7]. The basic helix-loop-helix leucine zipper transcriptional factor MITF has been shown to play a pivotal role in the development and differentiation of melanocytes, and can act as an oncogene as well in melanomas. While MITF expression in melanoma is variable across specimens [8], [9], studies have suggested that alterations to the repertoire of signals that determine MITF activity dictate the proliferative and invasive potential of melanoma cells [10], [11]. Disruptions in the MITF cascade, such as levels of the MITF regulator, BRAF, and the MITF target, c-Met, can lead to melanoma progression [7], [12], [13]. Moreover, recent studies have confirmed that miRNAs may have a role in the regulation of metastatic melanoma with alterations in the levels of the c-Met and MITF gene [10], [14].

Following its description in *C. elegans* in 1993, miRNAs are known to participate in essential biological processes through modulation of many mRNA transcripts and their subsequent protein progeny [15]. MiRNAs are endogenous, small RNAs that interfere with protein translation by binding target mRNAs; since its discovery, over 15,000 members have been identified [16]. MiRNAs, which can act as oncogenes and tumor suppressors, play a central role in tumorigenesis. For example, miR-15 and miR-16 can induce apoptosis by targeting the mRNA of the anti-apoptotic gene BCL2, which plays a key role in many types of human cancer, including leukemia, lymphoma and carcinoma [17]. Recently, miRNA expression has also been shown to be regulated by transcription factors. Studies revealed that miR-34a is a pro-apoptotic transcriptional target of the p53 tumor suppressor gene, with consequent effects on a variety of tumor types [14], [18], [19], [20]. In addition to miR-34a, p53 was found to regulate miR-182 expression in HCT116 colon cancer cells and H1299 lung cancer cells [20], [21].

miR-182, located between the c-Met and BRAF proto-oncogenes in the region of chromosome 7q31–34 [22], [23], is highly expressed in the retina [24]. The role of miR-182 in tumorigenesis, however, remains unclear. Previous studies have examined the function of miR-182 in BRAF dependent cutaneous melanomas [10]. We attempted to define the role of miR-182 in the development of BRAF independent posterior uveal melanomas. We examined the effect of miR-182 both *in vitro* and *in vivo* on cell proliferation and tumor growth. We also investigated targets of miR-182 including MITF, BCL2 and cyclin D2, identified through bioinformatic and functional assays. Altogether, miR-182 was found to function as a component of the p53 network and a tumor suppressor in posterior uveal melanoma cells.

## Results

### miR-182 Induction is Dependent on p53 Activation

**Figure 1:**
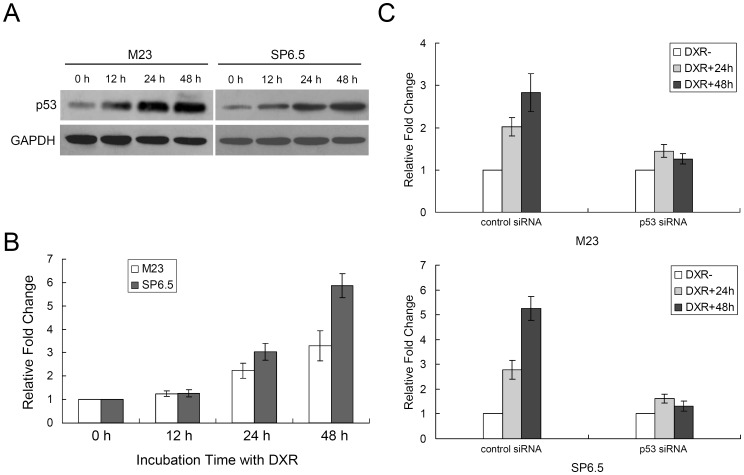
p53 induces miR-182 expression. (A) p53 expression levels were induced after treatment with doxorubicin (DXR, 1 µg/mL) in M23 and SP6.5 uveal melanoma cell lines. GAPDH was used as an internal control. (B) Induction of miR-182 expression after treatment with doxorubicin (1 µg/mL) in both cell lines. (C) M23 and SP6.5 cells transfected with a siRNA targeting p53 or a scrambled negative control siRNA were treated with 1 µg/mL of doxorubicin for 24 or 48 hours. miR-182 expression levels were indicated, as determined by real-time RT-PCR relative to the level of U6 snRNA expression. Representative data from three independent experiments are shown.

Since p53 can regulate cell growth through miRNA [20], we first sought to establish the relationship between p53 and miR-182. We observed that p53 started increasing at 12 hours, peaking at 24 hours, and persisted through 48 hours, after doxorubicin treatment in both M23 and SP6.5 cells (Fig. 1A). miR-182 expression was induced by incubating M23 and SP6.5 cells in the presence of doxorubicin. Quantitative real-time RT-PCR showed that miR-182 expression was increased in a time-dependent manner after doxorubicin treatment. For M23 cells, miR-182 showed a 2.20±0.32 fold increase at 24 hours and 3.30±0.65 fold increase at 48 hours. For SP6.5 cells, miR-182 showed a 3.01±0.37 fold increase at 24 hours and 5.80±0.52 fold increase at 48 hours (Fig. 1B). To determine if induction of miR-182 by doxorubicin is p53 dependent, we transfected M23 and SP6.5 cells with a siRNA that specifically targets p53 or a scrambled control siRNA. As shown in Fig. 1C, while the control siRNA did not change the induction of miR-182 by doxorubicin, p53 siRNA significantly reduced the expression levels of miR-182 in a dose-dependent fashion (Fig. S1).

### miR-182 Inhibits Cell Proliferation

**Figure 2:**
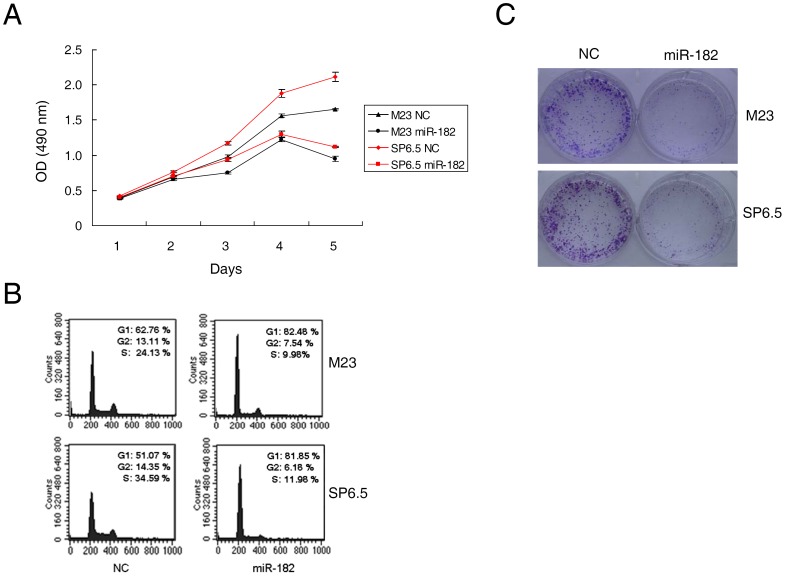
Ectopic miR-182 inhibits cell proliferation. (A) MTS assay was performed on days 1 to 5 as indicated after lipofectamine transfection of uveal melanoma cells M23 and SP6.5 with either miR-182 (50 nM) or a negative control scrambled oligonucleotide (NC). The data at each time point are expressed as the mean value ± SEM of the results obtained from triplicates in one experiment. Results represent those obtained in three independent experiments. (B) M23 and SP6.5 cells were collected 48 hours after transfection with miR-182 or NC, stained with propidium iodide, and analyzed by flow cytometry. Ten thousand cells were evaluated in each sample. The most representative results in three independent experiments are depicted. (C) M23 and SP6.5 cells transfected with miR-182 or NC were seeded at low density. After 7 days, colony formation was assessed by staining with crystal violet. Typical results from three independent experiments are shown.

The marked induction of miR-182 after p53 activation, prompted us to investigate whether miR-182 functioned as a tumor suppressor. The introduction of miR-182 caused a marked inhibition of cell proliferation in both M23 and SP6.5 cells compared with that of control miRNA (Fig. 2A). MTS assay was performed to assess growth inhibition between days 1 through 5 after transfection. The decrease in cell number was significant between cells transfected with miR-182 and cells transfected with a negative control at day 5 (42.81±3.61% decrease in M23 cells and 47.02±1.10% decrease in SP6.5 cells, p<0.01, n = 3). Complementary to the finding that miR-182 inhibited cell proliferation, miR-182 was found to cause increased G1 cell cycle arrest in these cells. M23 cells transfected with miR-182 showed 82.48% G1 arrest in comparison to 62.76% for negative control. SP6.5 cells transfected with miR-182 showed 81.85% G1 arrest in comparison to 51.07% with negative control (Fig. 2B). Furthermore, ectopic expression of miR-182 dramatically suppressed colony formation as depicted by crystal violet staining after 7 days of culture following transfection (Fig. 2C).

### miR-182 Inhibits Cell Migration and Invasion

**Figure 3:**
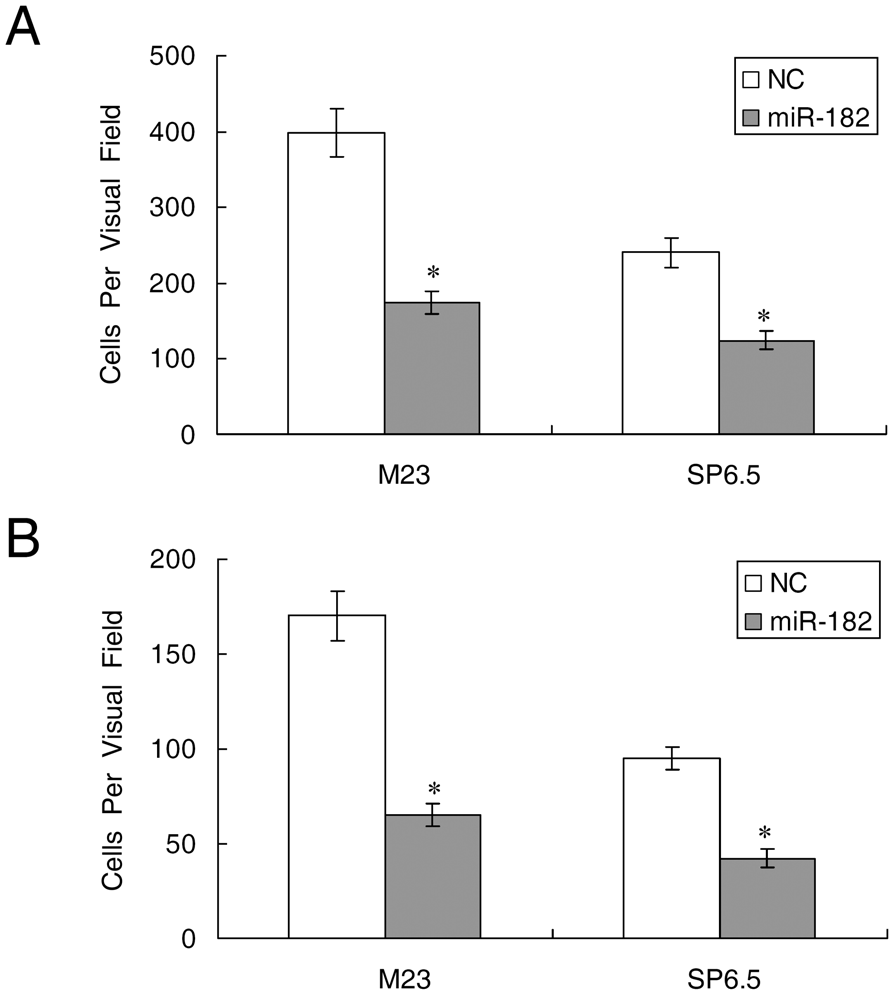
Ectopic miR-182 inhibits cell migration and invasion. Uveal melanoma cells M23 and SP6.5 were transfected with miR-182 or a negative control (NC) for 24 hours and plated on either culture or Matrigel inserts. The number of cells that had migrated through the culture insert pores (A) or Matrigel insert pores (B) was quantified by counting five independent visual fields using a 20X microscope objective. Results represent those obtained in three experiments. *: Differences in cell migration or invasion between miR-182 and negative control transfected cells were significant, p<0.01.

M23 and SP6.5 cells transfected with miR-182 were assessed for their ability to migrate and invade through transwell experiments. As shown in Fig. 3A, the HGF-induced migration was significantly decreased when comparing miR-182 transfected cells to negative control transfected cells (174±15 vs. 398±32 in M23 cells, and 124±12 vs. 236±20 in SP6.5, p<0.01, n = 3). Fig. 3B showed that HGF-induced invasiveness was also significantly hampered following miR-182 transfection (65±6 vs. 170±13 in M23 cells, and 42±5 vs. 95±6 in SP6.5, p<0.01, n = 3).

### miR-182 Enhances Apoptotic Activity and Cell Death

**Figure 4:**
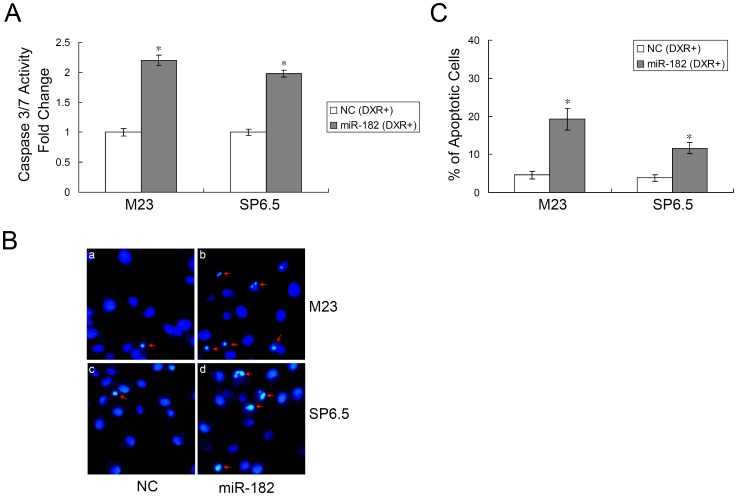
miR-182 enhances apoptosis. (A) Caspase 3/7 activity assay was performed on M23 and SP6.5 cells transfected with either miR-182 or a negative control (NC) after doxorubicin (DXR, 1 µg/mL) treatment. Relative caspase 3/7 activity is indicated in comparison to negative control. Results are expressed as the mean value ± SEM of the results obtained from triplicates in one experiment. Results represent those obtained in three experiments. (B) M23 and SP6.5 cells (miR-182 and NC transfected) were treated with doxorubicin for 48 hours. Cells were then stained with Hoechst 33342 and examined using a 20X fluorescent microscope objective. (C) M23 and SP6.5 cells with both condensed and fragmental nuclei were counted manually following treatment with doxorubicin as described above. Results represent those obtained in three experiments. *: Differences in apoptotic cell number between miR-182 and negative control transfected cells were significant, p<0.01.

After 48 hours, caspase 3/7 activity was significantly increased in miR-182 transfected cells in comparison to negative control after doxorubicin treatment. M23 cells demonstrated a 2.20±0.08 fold increase in caspase activity and SP6.5 cells demonstrated a 1.98±0.06 fold increase in caspase activity (Fig. 4A). Cell death was quantified using Hoechst staining. After treatment with doxorubicin, miR-182 transfected cells showed diminished viability and higher incidence of DNA fragmentation as well as chromatin condensation (Fig. 4B & C). M23 cells transfected with miR-182 had 19.23±2.87% apoptotic cells versus 4.60±1.03% apoptotic cells in negative control transfected cells (p<0.01, n = 3). SP6.5 cells transfected with miR-182 had 11.65±1.44% apoptotic cells versus 3.82±0.89% apoptotic cells in negative control transfected cells (p<0.01, n = 3). Similar results were obtained using the TUNEL assays (data not shown).

### MITF, BCL2, and Cyclin D2 are Targets of miR-182

**Figure 5:**
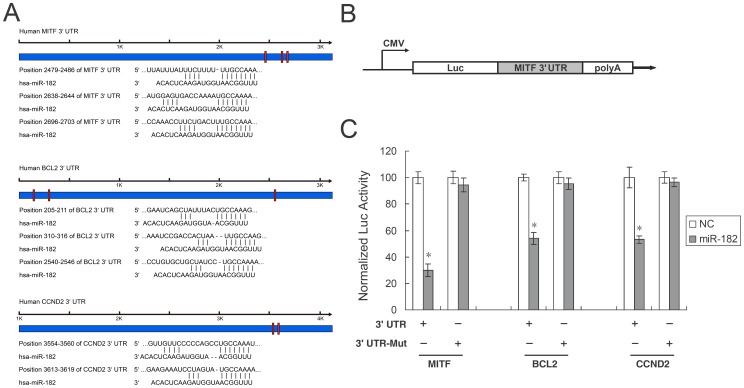
MITF, BCL2 and cyclin D2 (CCND2) are targets of miR-182. (A) Specific locations of the binding sites were marked with red color and MITF, BCL2 and cyclin D2 3′ UTR were marked with blue color. Alignment between the predicted miR-182 target sites and miR-182, the conserved 7–8 bp “seed” sequence for miR-182:mRNA pairing is indicated. (B) Diagram depicting the pMIR luciferase reporter constructs, containing a CMV promoter, which was utilized to verify the putative miR-182 binding sites. (C) HEK293 cells were co-transfected with miR-182, pLuc-target 3′ UTR, or a mutant 3′ UTR, along with a pRL-SV40 reporter plasmid. After 24 hours, the luciferase activity was measured. Values are presented as relative luciferase activity after normalization to *Renilla* luciferase activity. As shown, only in the presence of both miR-182 and the normal pLuc-MITF 3′ UTR (or equivalent) was there suppression of luciferase activity. Results represent those obtained in three separate experiments. *: Differences in luciferase activity between miR-182 and negative control transfected cells were significant, p<0.01.

To identify target genes of miR-182, we searched public databases, TargetScan (http://www.targetscan.org). Predicted target genes whose downregulation may mediate the biological effects of miR-182 include MITF, BCL2, and cyclin D2. Only MITF has previously been validated as subject to control by miR-182 during the development of the mouse retina [24]. We found by bioinformatic analysis that the MITF 3′ UTR contains three target sequences for miR-182 at positions 2479–2486, 2638–2644, and 2696–2703. BCL2 contains three target sequences at positions 205–211, 310–316, and 2540–2546. Cyclin D2 contains two target sequences at positions 3554–3560 and 3613–3619 (Fig. 5A). In order to test if miR-182 directly targets MITF, BCL2, and cyclin D2 genes, we cloned the wildtype 3′ UTR of each gene into a luciferase reporter vector, exemplified by Fig. 5B. We then transfected each resulting reporter construct (pLuc-MITF 3′ UTR, pLuc-BCL2 3′ UTR, and pLuc-CCND2 3′ UTR) into HEK293 cells, along with miR-182 or a negative control miRNA. The luciferase activity assays at 24 hours post-transfection demonstrated that miR-182 suppressed luciferase reporter activity to 30.00±4.70%, 54.00±4.40%, and 53.20±2.85% using 3′ UTR of MITF, BCL2 and cyclin D2, respectively (Fig. 5C). To demonstrate the specificity of miR-182 against the MITF, BCL2 and cyclin D2 genes, we generated mutation reporter constructs of each of the three genes and examined if these mutations would eliminate the suppression of the luciferase reporter activity. Fig. 5C demonstrated that mutations of all the sites attenuated the suppression of luciferase reporter activity by miR-182. These experiments demonstrate that miR-182 directly targets the MITF, BCL2, and cyclin D2 genes through their 3′ UTR, and the binding sites of miR-182 on MITF, BCL2, and cyclin D2 3′ UTR are required for its suppressive activity.

### Introduction of miR-182 Downregulates the Expression of MITF which in Turn Downregulates c-Met

**Figure 6:**
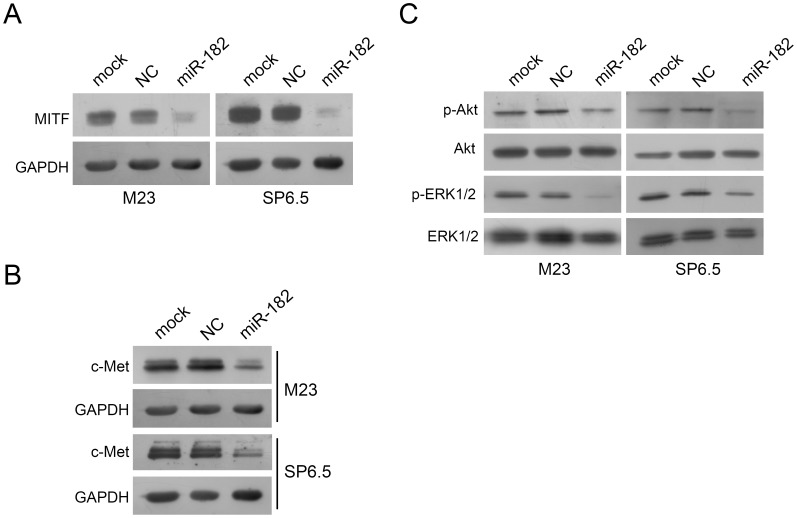
miR-182 downregulates the expression of MITF and c-Met. (A) MITF expression in M23 and SP6.5 cells after transfection with miR-182 was dramatically reduced by Western blot analysis. GAPDH was used as an internal control. (B) c-Met expression in M23 and SP6.5 cells after transfection with miR-182 was dramatically reduced by Western blot analysis. GAPDH was used as an internal control. (C) miR-182 downregulated expression of p-Akt and p-ERK1/2, but not total Akt or ERK1/2. M23 and SP6.5 cells were either mock, transfected with a negative control or miR-182, lysed, and then used for Western blot analysis.

Western blot analysis showed that MITF was indeed dramatically reduced when cells were transfected with miR-182 (Fig. 6A). c-Met, which is a target of MITF, was decreased in M23 and SP6.5 cells transfected with miR-182 in comparison to either mock or a negative control transfected cells (Fig. 6B). c-Met has been shown to activate diverse intracellular signaling pathways including Akt and ERK1/2 [25]. As shown in Fig. 6C, downregulation of c-Met by miR-182 led to a significant reduction of phosphorylated-Akt and phosphorylated-ERK1/2 in both M23 and SP6.5 cells. Both total Akt and total ERK1/2 were not affected when comparing miR-182 transfection to negative control transfection (Fig. 6C).

### Downregulation of c-Met Inhibits Uveal Melanoma Cell Proliferation, Migration and Invasion

**Figure 7:**
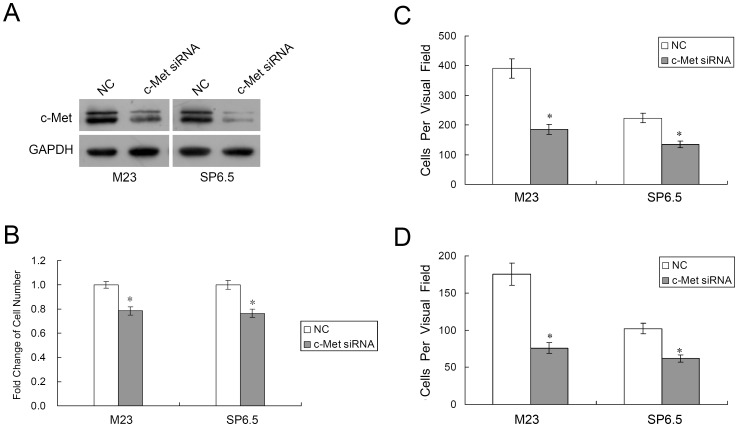
Downregulation of c-Met suppresses uveal melanoma cell proliferation, migration and invasion. (A) Western blot analysis was performed to detect c-Met expression after lipofectamine transfection of uveal melanoma cells M23 and SP6.5 with either c-Met siRNA (50 nM) or a negative control (NC). (B) MTS cell proliferation assay after 3 days is shown. The data are expressed as the mean value ± SEM of the results obtained from triplicates in one experiment. Results represent those obtained in three experiments. (C&D) Uveal melanoma cells M23 and SP6.5, transfected with c-Met siRNA or NC, were quantified for migratory (C) or invasive (D) studies using culture or Matrigel inserts. Results represent those obtained in three experiments. *: Differences in cell migration or invasion between c-Met siRNA and negative control transfected cells were significant, p<0.01.

Since c-Met is a direct target of MITF, we also investigated the effect of c-Met on uveal melanoma cells. c-Met specific siRNA was used to decrease the expression of c-Met in both M23 and SP6.5 cells (Fig. 7A). MTS assays showed that transfection of c-Met siRNA caused a dramatic inhibition of M23 and SP6.5 cell growth at 72 hours (21.58±3.28% decrease in M23 cells and 23.47±3.46% decrease in SP6.5 cells, p<0.01, n = 3; Fig. 7B). Also, as shown in Fig. 7C, HGF-induced migration was significantly decreased when comparing c-Met siRNA transfected cells to negative control transfected cells (185±17 vs. 391±33 in M23 cells, and 131±11 vs. 223±16 in SP6.5, p<0.01, n = 3). Fig. 7D showed that HGF-induced invasiveness was also significantly hampered following c-Met siRNA transfection (76±7 vs. 175±15 in M23 cells, and 62±5 vs. 102±7 in SP6.5, p<0.01, n = 3).

### Introduction of miR-182 Downregulates Multiple Cell Signaling Pathways

**Figure 8:**
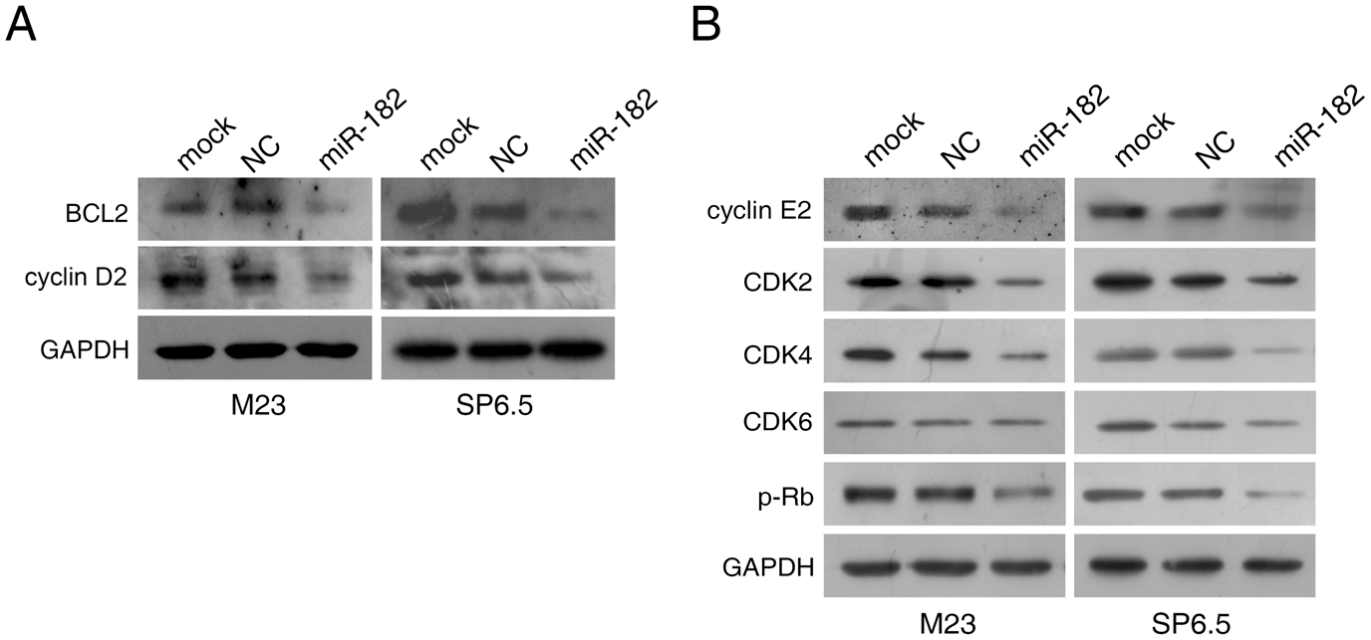
miR-182 downregulates multiple cell signal pathways. Transfected M23 and SP6.5 cells were prepared and used for Western blot analysis with multiple antibodies. GAPDH was used as an internal control. (A) miR-182 downregulated expression of BCL2 and cyclin D2. (B) miR-182 downregulated cell cycle related proteins cyclin E2, CDK2, CDK4, and phosphorylated-Rb (p-Rb). Effect on CDK6 was not pronounced in M23 cells.

To examine other intracellular proteins affected by miR-182 in uveal melanoma cells, we next determined the expression patterns of intracellular proteins directly affected by miR-182, including BCL2, cyclin D2, and other cell cycle related proteins. Primary targets of miR-182 including BCL2 and cyclin D2 were suppressed by miR-182, as expected (Fig. 8A). Moreover, ectopic miR-182 delivery also downregulated cell cycle regulatory proteins such as cyclin E2, CDK2, CDK4, and phosphorylated-retinoblastoma protein (p-Rb) in both M23 and SP6.5 cells (Fig. 8B). CDK6 levels were less markedly affected (Fig. 8B).

### Overexpression of miR-182 Suppresses the Growth of Uveal Melanoma Cells *in vivo*

**Figure 9:**
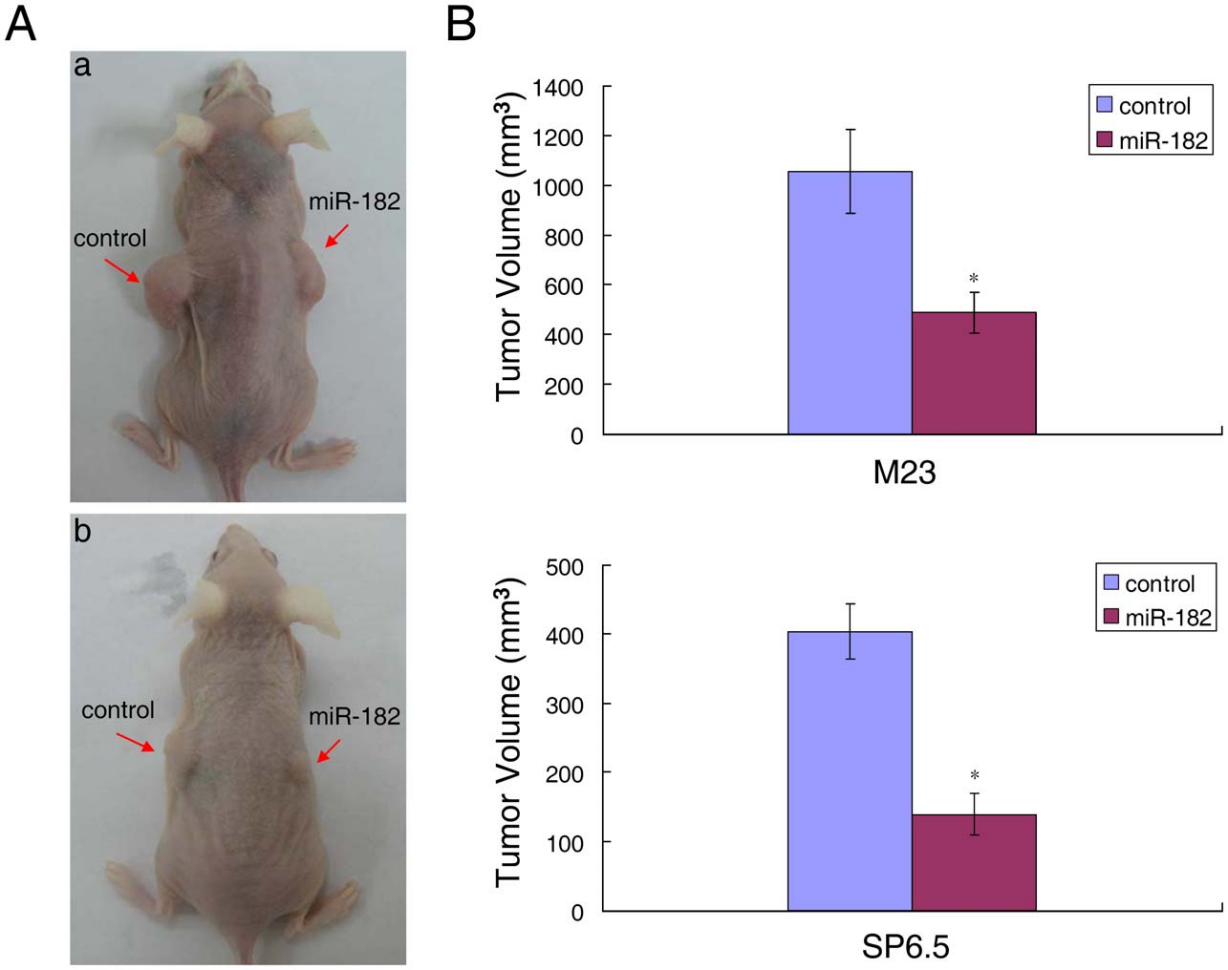
miR-182 suppresses uveal melanoma cell growth *in vivo*. (A) Representative photographs of nude mice 4 weeks after inoculation with miR-182 or control lentivirus infected uveal melanoma cells. a: Inoculation with M23 cells; b: Inoculation with SP6.5 cells. (B) Average volume of tumors derived from M23 or SP6.5 cells infected with miR-182 or control lentivirus in nude mice. *: Differences in tumor volume between miR-182 and control infected cells were significant, n = 4 each, p<0.05 for M23 cells inoculation, p<0.01 for SP6.5 cells inoculation.

Since miR-182 inhibited the proliferation and migration of uveal melanoma cells *in vitro*, we next investigated if overexpression of miR-182 could suppress tumor cell growth *in vivo*. M23 and SP6.5 cells were infected with lentivirus expressing miR-182 or a negative control. The infected cells were then injected subcutaneously into the flanks of nude mice. After 4 weeks, the averaged tumor volumes were significantly lower in cells infected with lentivirus expressing miR-182, as compared with control (Fig. 9). The results demonstrate that overexpression of miR-182 suppresses the growth of uveal melanoma cells *in vivo* as well.

## Discussion

Aberrant regulation of miRNAs has been implicated in human uveal melanoma development [14], [26]. Downregulation of subsets of miRNAs is a common finding in certain cancers, suggesting that some of these miRNAs may act as putative tumor suppressor genes. In this study, we identified miR-182 as another miRNA that is frequently downregulated in human uveal melanoma (Fig. S2). miR-182 serves as a doxorubicin-responsive miRNA that regulates uveal melanoma cell growth in a p53-dependent manner (Fig. 1). Specifically, we demonstrated that introduction of miR-182 inhibited cell proliferation, migration and invasion. Target prediction and *in vitro* functional studies showed that MITF is a direct target of miR-182 (Fig. 5A & C), as supported by previous studies [10], [24].

MITF is a tissue specific transcription factor that is essential for melanocyte development [7]. Functionally, MITF serves as a master regulator of melanocyte development and a melanoma oncogene [7]. Melanocytes, which are cells of the neural crest origin, can give rise to the pigmented cells of the epidermis and the uvea [7]. Recently, studies of aberrant miR-182 expression demonstrated that miR-182 promotes cutaneous melanoma metastasis by suppressing the transcription factors FOXO3 and MITF [10]. More intriguingly, Segura et al. showed that enhanced miR-182 expression and MITF downregulation is associated with advanced stage of cutaneous tumor progression [10], [27], [28], [29]. This is in contrast to findings by Schultz et al. and Caramuta et al., who both found miR-182 to be downregulated in melanoma samples [30], [31]. Garraway et al., through integrative genomic analyses, identified MITF as an oncogene amplified in melanoma and correlated with decreased overall patient survival [32]. Furthermore, MITF amplification is associated with BRAF mutation and p16 inactivation [32]. The divergent views, though contradictory, belies the differences between cutaneous melanomas and posterior uveal melanomas. Although epidermal melanoma and uveal melanoma share common origins and carry with them histological and nomenclatural similarities, their clinical behavior and intracellular regulation are very different. Our findings in this study support the different biological effects of MITF on cutaneous melanomas and posterior uveal melanomas. The p53 pathway, vital in many tumor oncogenesis, appears intact in posterior uveal melanomas as opposed to cutaneous melanomas [14]. The posterior uveal tract consists of the choroidal and ciliary body with melanization of the tissue remaining dormant following birth. In contrast, epidermal melanocytes showed continued melanin turnover throughout the life of the organism [33]. Positive expression of MITF, however, remains in most adult clinical uveal melanoma specimens [33]. Concordant with these findings, MITF in choroidal melanoma behaves as an oncogenic factor that is suppressed by miR-182 as demonstrated in this study, as well as by miR-137 in our previous report [34]. These studies explain the differential expression patterns of MITF in choroidal and cutaneous melanoma and substantiate the claim that MITF serves as a regulator of melanocyte development and melanoma oncogenesis [7], [10], [33], [34]. Taken together, we surmise that MITF causes cellular proliferation and oncogenesis in most posterior uveal melanomas and some cutaneous melanomas with MITF amplification.

In this study, miR-182 was shown to inhibit cell proliferation and migration by regulating the expression of MITF, which in turn downregulated c-Met expression (Fig. 6). Mechanistically, MITF plays an essential role in the homeostatic upregulation of c-Met expression by direct binding to the c-Met promoter in melanoma cells and primary melanocytes [35]. c-Met, which is highly expressed in melanomas, is thought to account for the metastatic potential of melanomas (Figure 7) [36], [37]. Furthermore, c-Met mediated inhibition of the Akt and ERK1/2 pathways can lead to cell cycle arrest (Fig. 2B). Another mechanism of MITF in cell cycle regulation could be attributed to its modulation of CDKs. Regulation of CDK2 by MITF is essential for melanoma clonogenic growth [38]. A tight correlation has been observed between MITF and CDK2 expression levels in primary melanoma specimens and several melanoma cell lines [38]. CDK4, a melanoma susceptibility gene, has also been shown to be a target of MITF [13]. Consistent with those observations, our results showed that CDK2 and CDK4 were downregulated in cells with suppression of MITF after introduction of miR-182 (Fig. 8).

Globally, under the directions of the p53 tumor suppressor network, miR-182 regulates cell proliferation, apoptosis, migration and invasion. Part of the effects of miR-182 is contingent upon the presence of MITF, which in turn regulates the HGF/c-Met signaling pathway. This study also found that miR-182 can inhibit BCL2 and cyclin D2 directly (Fig. 5A, C). BCL2, which is an anti-apoptotic gene, is also influenced by the presence of MITF [39]. Cyclin D2, as a positive regulator of G1 phase cell cycle progression, is shown to be a direct target of miR-182 in this study (Fig. 5A & C). The downregulation of miR-182 in uveal melanoma specimens suggests that miR-182 may be, at least in part, responsible for the development of uveal melanoma. The various effects of miR-182 as demonstrated in this study, both *in vitro* and *in vivo*, will hopefully help illuminate the mechanisms behind uveal melanoma oncogenesis.

## Materials and Methods

### Cell Culture

The human uveal melanoma cell lines M23 and SP6.5 were isolated from Caucasian patients with primary choroidal melanoma and grown in Dulbecco modified Eagle’s media (DMEM; Invitrogen, Carlsbad, CA) supplemented with 10% fetal bovine serum (FBS; Hyclone, Logan, UT) as described [40]. HEK-293 cells, purchased from ATCC (Manassas, VA), were cultured under the same conditions.

### Ethics Statement

This study was carried out in strict accordance with the recommendations and approval of the Wenzhou Medical College Animal Care and Use Committee (Permit Number: WZMCOPT-050809). All the human uveal melanoma specimens were obtained from patients treated at the Eye Hospital, Wenzhou Medical College (Wenzhou, China). Sample collection was approved by the Wenzhou Medical College Ethics Committee on research involving human subjects, and written informed consent was obtained from each case. All experiments were performed in compliance with the Helsinki Declaration and national laws.

### Quantitative RT-PCR

Total RNA was extracted from cells with Trizol reagent (Invitrogen) and the integrity was confirmed using spectrophotometry and formaldehyde/agarose gel electrophoresis. 10****ng of total RNA were used for cDNA synthesis by the Taqman® MicroRNA Reverse Transcription Kit (Applied Biosystems, Foster City, CA), and miR-182 expression level was quantified by the Taqman MicroRNA Assay (Applied Biosystems), according to manufacturer’s instructions. Real-time RT-PCR was performed using the Applied Biosystems 7500 Fast Real-Time PCR System (Applied Biosystems), the relative expression level of miR-182 was normalized to the U6 snRNA and calculated as previously reported [41].

### Cell Proliferation Assay

M23 and SP6.5 cells were seeded at 3×10^3^ cells per well in 96-well plates (Costar, High Wycombe, UK) for each transfection. For each well, 50 nM of miR-182 precursor molecule (Ambion, Austin, TX) or a negative control precursor miRNA (Ambion) was transfected using Lipofectamine 2000 (Invitrogen). After 24-hour culture, cell proliferation was assessed using the CellTiter 96 AQ_ueous_ assay kit (Promega, Madison, WI) according to the manufacturer’s instructions. c-Met specific siRNA (Ambion) and negative control siRNA (Ambion) were used to downregulate c-Met expression in uveal melanoma cells. 50 nM of c-Met specific siRNA or negative control siRNA was transfected into M23 and SP6.5 cells with Lipofectamine 2000. MTS assay was carried out 72 hours after transfection, as described above.

### Flow Cytometry Analysis

Forty-eight hours after transfection with 50 nM of miRNAs, M23 and SP6.5 cells (1×10^5^) were stained with propidium iodide using the Cycle Test Plus DNA Reagent Kit (BD Biosciences, San Jose, CA) and then analyzed for DNA content with a flow cytometry (FACScaliber; BD Biosciences).

### Transwell Migration and Matrigel Invasion Assays

M23 and SP6.5 cells were grown to ∼ 60% confluence, transfected with 50 nM of miRNAs, and harvested by trypsinization in 24 hours. To measure cell migration, 8 mm pore size culture inserts (Transwell; Costar) were placed into the wells of 24-well culture plates. To measure cell invasion, 8 mm pore size Matrigel inserts (Becton Dickinson, Mountain View, CA) were used. The Matrigel inserts were rehydrated prior to use. In both experiments, 400 µL of DMEM containing recombinant human hepatocyte growth factor (HGF, 20 ng/mL; R&D Systems, Minneapolis, MN) were added in the lower chamber. 1×10^5^ cells were then seeded in the upper chamber. After 24 hours of incubation, the number of cells that had migrated was quantified by counting 10 independent visual fields (Zeiss, Oberkochen, Germany) using a 20X objective. Similarly, M23 and SP6.5 cells were analyzed for migration and invasion after transfection with 50 nM of c-Met specific siRNA or negative control siRNA.

### Hoechst Staining

Forty-eight hours after transfection with 50 nM of each miRNA, doxorubicin (1 µg/mL; Sigma, St. Louis, MO) was added to each cell type (M23 and SP6.5 cells) prior to staining with Hoechst 33342 (5 µg/mL, Sigma) to visualize both condensed and fragmented nuclei. After 20 minutes of staining at 25°C, cells were examined under fluorescence microscope (Zeiss) using a 20X objective.

### Caspase Activity Assay

Apoptosis in M23 and SP6.5 cells was determined using the Caspase-Glo 3/7 Assay kit (Promega) according to manufacturer’s instructions. Transfected cells were first treated with doxorubicin (1 µg/mL) for 48 hours, lysed, and then incubated with the caspase substrate for 2 hours, followed by reading by a microtiter plate reader (Molecular Devices, Sunnyvale, CA).

### Luciferase Reporter Assays

The 3′ untranslated region (UTR) of human MITF, BCL2 or cyclin D2 was amplified from human genomic DNA and individually cloned into pMIR-REPORT vector (Ambion) by directional cloning. Seed regions were mutated to remove all complementarity to nucleotides 1–7 of miR-182 using the QuickchangeXL Mutagenesis Kit (Stratagene, La Jolla, CA). HEK-293 cells were co-transfected with 0.4 µg of firefly luciferase reporter vector and 0.02 µg of the control vector containing *Renilla* luciferase, pRL-SV40 (Promega), using Lipofectamine 2000 (Invitrogen) in 24-well plates (Costar). Each transfection was performed in four wells. For each well, 50 nM of miRNAs was co-transfected with the reporter construct as indicated in Figure 5. Luciferase assays were carried out 24 hours after transfection using the Dual Luciferase Reporter Assay System (Promega). Firefly luciferase activity was normalized to *Renilla* luciferase activity.

### Western Blot Analysis

M23 and SP6.5 cells (1×10^5^) were seeded and grown for 24 hours, transfected and subjected to lysis in a lysis buffer (50 mM/L Tris-Cl, 1 mM/L EDTA, 20 g/L sodium dodecyl sulfate [SDS], 5 mM dithiothreitol, 10 mM phenylmethylsulfonyl fluoride). Equal amounts of protein lysates (50 µg each) and rainbow molecular weight markers (GE Healthcare Life Sciences, Piscataway, NJ) were separated by 10% SDS-polyacrylamide gel electrophoresis (PAGE), then electrotransferred to nitrocellulose membranes. The membranes were blocked with a buffer containing 5% non-fat milk in PBS with 0.05% Tween 20 for 2 hours and incubated overnight with antibody at 4°C. After a second wash with PBS containing 0.05% Tween 20, the membranes were incubated with peroxidase conjugated secondary antibodies (Santa Cruz Biotechnology, Santa Cruz, CA) and developed with an electrogenerated chemiluminescence (ECL) detection kit (Pierce, Rockford, IL). Antibodies for cyclin D2, BCL2, cyclin E2, CDK2, CDK4, CDK6, total ERK1/2, phosphorylated-ERK1/2, total Akt, phosphorylated-Akt, and phosphorylated-Rb were from Cell Signaling Technology (Beverly, MA), MITF was from Calbiochem (San Diego, CA), and c-Met was from Santa Cruz Biotechnology.

### *In vivo* Tumor Growth Assay

The pre-miRNA expression construct lenti-miR-182 and pCDH-CMV-MCS-EF1-copGFP control vector were purchased from System Biosciences (Mountain View, CA). The lentivirus was produced according to the manufacturer’s instructions. M23 cells and SP6.5 cells were infected with lentivirus expressing miR-182 or a negative control. Female nude mice at 6 weeks of age were used for xenograft studies. M23 cells (5×10^6^) or SP6.5 cells (5×10^6^) expressing miR-182 or the negative control were inoculated subcutaneously into the flanks of nude mice. All mice were sacrificed 4 weeks later. Tumor size was measured with a caliper, and the volume was calculated using the formula: (*L*×*W* ^2^) ×0.5, (*L*, length; *W*, width) [42].

### Analysis of miR-182 Expression in Uveal Melanoma Clinical Samples

To extract RNA from formalin-fixed paraffin-embedded (FFPE) uveal melanoma, 8 µm sections were cut from FFPE samples using a microtome (Leica RM2135, Leica Microsystems, Wetzlar, Germany). LCM (laser capture microdissection) was carried out on each FFPE sample to specifically capture normal uveal melanocytes from uveal tissues as well as uveal melanoma cells from tumor tissues [43]. The whole procedure was conducted by the Applied Biosystems® Arcturus*^XT^*™ Microdissection System (Invitrogen), followed by total RNA extraction from the isolated cells, using the RecoverAll™ Total Nucleic Acid Isolation Kit (Ambion). Real-time RT-PCR was then performed to examine the expression level of miR-182 in the samples.

### Statistical Analysis

All data were shown as the mean ± SEM. Differences between cells transfected with miR-182 and a negative control were analyzed using the Student’s *t*-test. Statistical significance was accepted at p<0.05.

## Supporting Information

Figure S1 **p53 induces miR-182 expression in uveal melanoma cells in response to doxorubicin (DXR) treatment.** M23 and SP6.5 cells transfected with a siRNA targeting p53 or a negative control siRNA at different doses were treated with 1 µg/mL of doxorubicin for 48 hours. miR-182 expression levels were indicated, as determined by real-time RT-PCR relative to the level of U6 snRNA expression.
(TIF)

Figure S2 **miR-182 expression is downregulated in human uveal melanoma specimens.** Real-time RT-PCR analysis was performed to detect the expression of miR-182 in uveal melanoma clinical samples. miR-182 was significantly decreased in tumor specimens as compared with normal uveal melanocytes from uveal tissues, except in samples 5 and 6. The expression of miR-182 in uveal melanocytes was set at 1, and the relative expression level of miR-182 in tumors was shown as fold change. U6 snRNA was used as an internal control. N: normal uveal melanocytes from uveal tissues; T: tumor tissues. Results represent those obtained in three experiments.
(TIF)
