# Supplementary figures and images for: Role of MicroRNA-182 in Posterior Uveal Melanoma: Regulation of Tumor Development through MITF, BCL2 and Cyclin D2
Source: PLoS One. 2012 Jul 27;7(7):e40967. doi: 10.1371/journal.pone.0040967 (PMC3407171; doi:10.1371/journal.pone.0040967)

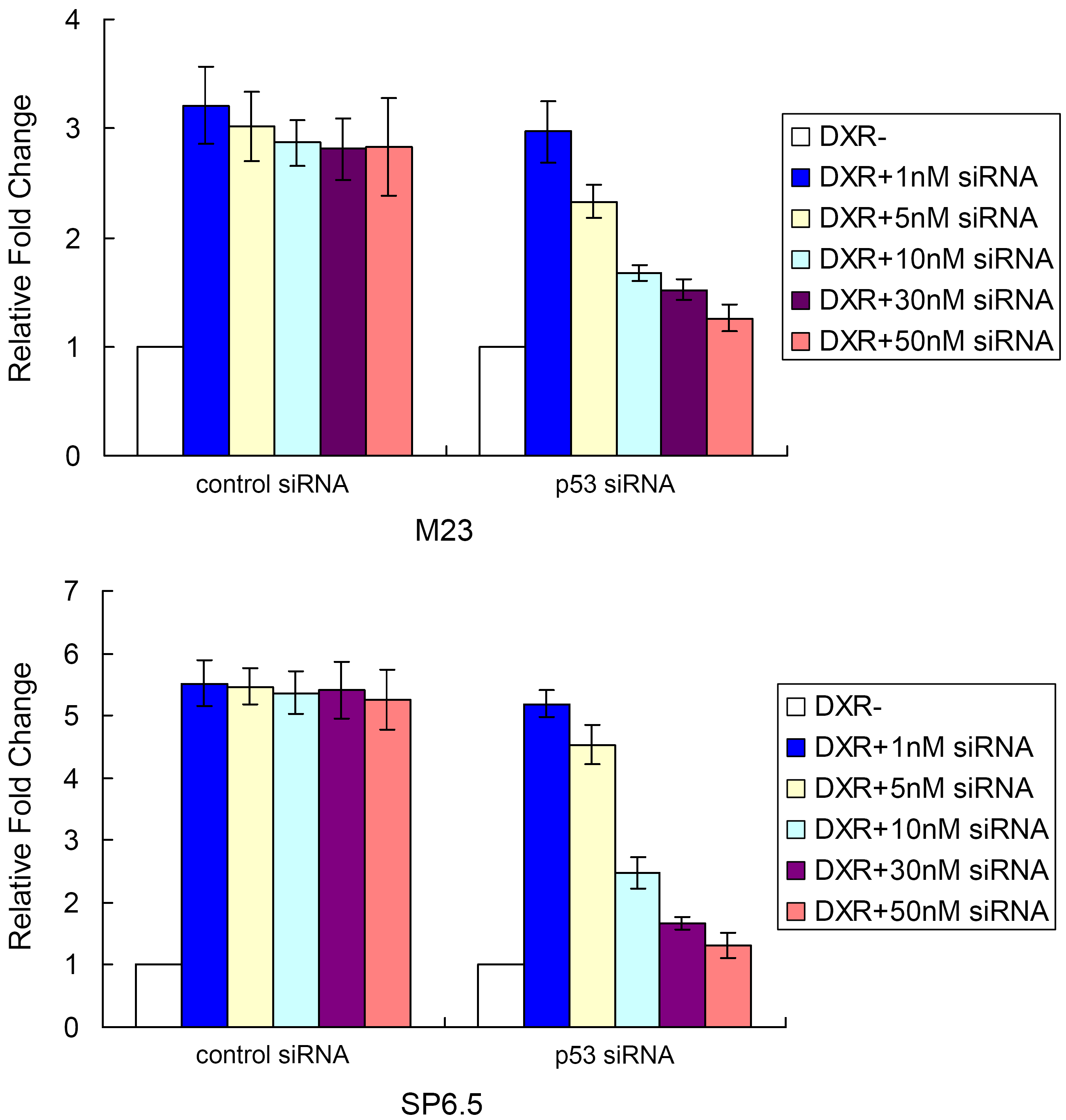

Supplement: Figure S1 — p53 induces miR-182 expression in uveal melanoma cells in response to doxorubicin (DXR) treatment. M23 and SP6.5 cells transfected with a siRNA targeting p53 or a negative control siRNA at different doses were treated with 1 µg/mL of doxorubicin for 48 hours. miR-182 expression levels were indicated, as determined by real-time RT-PCR relative to the level of U6 snRNA expression. (TIF) [file pone.0040967.s001.tif]

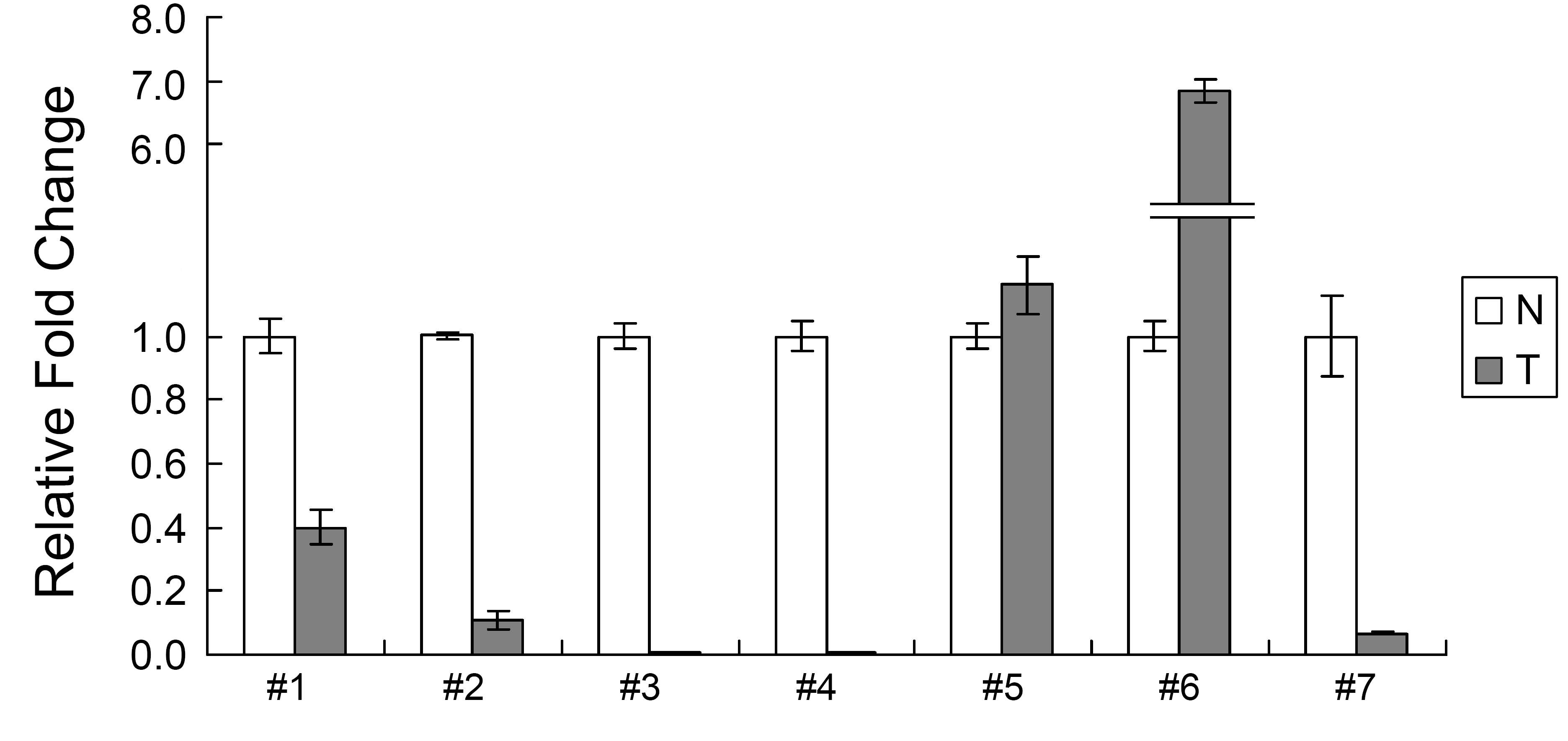

Supplement: Figure S2 — miR-182 expression is downregulated in human uveal melanoma specimens. Real-time RT-PCR analysis was performed to detect the expression of miR-182 in uveal melanoma clinical samples. miR-182 was significantly decreased in tumor specimens as compared with normal uveal melanocytes from uveal tissues, except in samples 5 and 6. The expression of miR-182 in uveal melanocytes was set at 1, and the relative expression level of miR-182 in tumors was shown as fold change. U6 snRNA was used as an internal control. N: normal uveal melanocytes from uveal tissues; T: tumor tissues. Results represent those obtained in three experiments. (TIF) [file pone.0040967.s002.tif]
